# Third Parties Mirror the Aggression of the Antagonists: A Video-Based Analysis of Third-Party Aggression in Interpersonal Conflicts

**DOI:** 10.1177/08862605211023503

**Published:** 2021-06-14

**Authors:** Peter Ejbye-Ernst, Marie Rosenkrantz Lindegaard, Wim Bernasco

**Affiliations:** 1 Netherlands Institute for the Study of Crime and Law Enforcement (NSCR), Amsterdam, Netherlands; 2 University of Amsterdam, Netherlands; 3 University of Copenhagen, Denmark; 4 Vrije Universiteit Amsterdam, Netherlands

**Keywords:** criminology, violence exposure, violent offenders

## Abstract

Third parties tend to take an active role and intervene in interpersonal conflicts in public. Previous research has shown that the level of aggression of these interventions determines how they influence the conflict. No previous study has, however, systematically investigated whether the aggression of third-party interventions is influenced by the development of the conflict situation. The objective of this study is twofold. First, the study determines the extent to which the aggression level of intervening third parties changes during the course of interpersonal conflicts. Second, the study identifies and investigates the factors that affect the aggression levels displayed by intervening third parties. We systematically observed and coded CCTV footage of 46 interpersonal conflicts in public space, recorded by surveillance cameras in Amsterdam, the Netherlands. The data included 565 intervention behaviors by 125 third parties. We recorded the levels of aggression of the individuals involved in the conflict and conducted a multinomial logistic regression analysis to investigate what influenced the aggression level of the third-party interventions. We found that the aggression levels of the preceding intervention behaviors by the third parties predict aggression levels of their subsequent interventions. This shows a consistency in third-party interventions over the course of a conflict. We also found that the aggression levels of the conflict parties that are the targets of the interventions influence the aggression levels of third-party intervention. This finding demonstrates that the development of the conflict situation influences how aggressive the third parties are. Our study emphasizes the importance of taking the interactional dynamics of interpersonal conflicts into consideration when explaining third-party behavior.

## Introduction

A number of empirical studies have found that third parties are present and actively intervene in a large proportion of real-life assaults and interpersonal conflicts (Felson et al., 1984; Philpot et al., 2018; Planty, 2002; Wells & Graham, 1999). These interventions are, however, not a uniform phenomenon. Intervention behaviors span from calm mediators gesturing softly to third parties that act as partisans and join a conflict as reinforcements to one of the antagonists (Black & Baumgartner, 1983; Phillips & Cooney, 2005). Some interventions are thus mild and nonaggressive, whereas others are physically forceful, or even violent.

Previous literature has found that the level of aggression of an intervention behavior is a key factor in explaining the impact it has on the conflict development. While nonaggressive interventions tend to decrease the violence of a conflict, more aggressive interventions seem to have the opposite effect (Levine et al., 2011; Parks et al., 2013; Phillips & Cooney, 2005). Third parties thus appear Janus-faced: On the one hand they hold the potential to reduce the severity or even end conflicts, but on the other hand they pose a risk of escalation as they might join the fight and turn it into a group brawl (Levine et al., 2011; Wells & Graham, 1999). Knowing what makes a third-party intervene at a specific level of aggression is thus of the utmost importance if we want to understand how interventions by third parties impact the trajectory of interpersonal conflicts.

While the literature on third-party aggression thus finds that the aggressiveness of third-party interventions influences the development of an interpersonal conflict, it typically assumes this influence to be unidirectional. Felson et al. (1984, p. 457), e.g., write that they assume “third parties influence the offender and victim and not the reverse.” Recent empirical studies, however, indicate that third parties are not necessarily consistent in their intervention manners and sometimes change the level of aggression of their intervention throughout the situation (Levine et al., 2011; Liebst et al., 2019a).

This change in the intervention behavior indicates that something within the situation makes the third parties change in aggressiveness. Since previous research finds the aggressiveness of third-party interventions plays a key role in the overall severity of the conflict (Levine et al., 2011; Parks et al., 2013; Phillips & Cooney, 2005), it is essential to investigate which situational developments make the third parties change their behavior. Furthering the understanding of third-party intervention is thus furthering the understanding of the dynamics that lead to interpersonal violence or prevent it.

This article investigates the aggression of third-party intervention in two steps. First, the article explores whether a behavioral analysis of the sequences of third-party intervention behaviors corroborates the finding from the observational studies that some third parties intervene at varying levels of aggression throughout a conflict situation, i.e., that they are not always consistent. Second, the study investigates whether the development of the conflict situation can explain the changes in aggression of third-party interventions. In order to do this, we carry out a systematic behavioral analysis of CCTV footage of real-life conflicts from the streets of Amsterdam. First, we find that even though consecutive intervention behaviors are mostly at the same level of aggression, third parties sometimes change their level of aggression. Second, we find that an increase in the number of violent behaviors performed by the antagonist targeted with the intervention behavior significantly increases the chance that an intervention behavior will be more aggressive. This finding indicates that third parties respond to the behavior of antagonists by mirroring the aggressiveness of the individual they target, which in turn could lead to a polarization of interpersonal conflicts.

### Consistency or Adaptation of Third Parties

The scientific literature typically typologizes third parties into mutually exclusive roles or categories. These typologies have been given a multitude of names, such as: *aggressive* vs. *nonaggressive* (Wells & Graham, 1999), *mediator* vs. *partisan* (Cooney, 1998), *mediate* vs. *engage* (Felson et al., 1984), and *surrogates* vs. *facilitators* vs. *precipitators* vs. *bystanders vs. incapable guardians* (Decker, 1995). While they differ in their definitions and the number of roles they identify, these typologies all share the assumption that a third party will fit one category for the duration of a conflict (note, however, that Decker (1995) specifies that third party roles are to be seen more as ideal types than as discrete categories). This assumption of consistency entails that a third party will not change his or her style of intervention during the conflict.

A possible explanation of this assumption of consistency of third-party behavior is that it is a product of the methodology used in the research. Researchers investigating third-party aggression have approached the subject with a number of empirical approaches, such as retrospective interviews (Phillips & Cooney, 2005), document analysis (Decker, 1995; Felson et al., 1984), naturalistic observation (Parks et al., 2013; Reynald, 2009), and observation of CCTV footage of conflicts (Levine et al., 2011; Liebst et al., 2019a). With the exception of the observation of CCTV footage, all of these approaches share the premise that they depend on the observer to record or recollect what happens throughout the conflict in real-time (Philpot et al., 2019). Since interpersonal conflicts are complex and typically erupt and end quickly, the reliability of recollection or real-time observation of interpersonal conflicts has been questioned (Collins, 2008; Philpot et al., 2019). This is especially pertinent in the study of third parties, since they are rarely awarded much attention in interpersonal conflicts: their behavior is rarely documented in official documents (Phillips & Cooney, 2005) and antagonists of conflicts have been found to have trouble recalling their presence (Bernasco et al., 2013). The assumed consistency of third-party behavior thus might be a methodological convenience to reduce the complexity researchers face carrying out real-time observations in the seemingly chaotic conflict situations.

This interpretation is substantiated through the findings of two studies that are based on CCTV footage. Out of the existing literature, these are the only studies that do not rely on observing or recalling the behavior in real time. The descriptive statistics of these studies indicate that most of the time third parties conform to performing either aggressive or nonaggressive behaviors. However, these studies also identify a number of third parties that performs *both* aggressive and nonaggressive intervention behaviors (Levine et al., 2011; Liebst et al., 2019a). This overlap between aggressive and nonaggressive behaviors contradicts the consistency assumption and begets the question of what engenders this change in the behavior of the third parties.

### The Social Context of Violence

In order to understand the role that behavior of third parties play in interpersonal conflicts, it is important to understand that violence is, like all other interpersonal behavior, constructed by the people present in the situation (Hepburn, 1973). As detailed in the introduction, these situations typically involve not only the antagonists of the conflict, but also a number of third parties. Within these situations, third parties can act in ways that create “*a definition of circumstances, actions, and individuals that enables violence to occur*” (Decker, 1995, p. 441). Third parties can influence the conflict development through their actions within the situation by promoting or discouraging violence. They can, e.g., try to mediate the conflict which allows the antagonists to back down without losing face or act aggressively themselves as adversaries of one of the conflict parties and escalate the conflict further.

The interactionist theory of violence (Tedeschi & Felson, 1994) describes how conflict behavior is a reaction to the previous behaviors in the situation. If we want to understand how individuals act, we should therefore look toward the previous behaviors within the conflict. This theoretical conceptualization of interpersonal conflicts insists that, while genetics and previous personal experiences might be central in selecting who gets involved in a conflict, we must also look toward the behavior of other people in the conflict to understand how a conflict develops (Felson et al., 1984; Felson. & Tedeschi, 1993; Jackson-Jacobs, 2013; Luckenbill, 1977; Tedeschi & Felson, 1994).

The interactionist theory has previously been used to investigate how the antagonists of interpersonal conflicts influence each other (Felson & Tedeschi, 1993; Luckenbill, 1977). In this article, we use this theory to understand the interventions of third parties. We propose that the changes in aggression of third-party intervention behaviors are reactions to the behavior of the antagonists in the situation. The aggressiveness of the antagonists in a conflict situation might influence the aggressiveness of third-party interventions in three different ways:

First, third parties might use aggression as a means to stop the aggression of an antagonist (Levine et al., 2011). In this case, the intervening third-party uses aggression not as a goal in itself, but rather as a tool to change the trajectory of the conflict situation (Tedeschi & Felson, 1994). If a third party wants to stop a very violent antagonist, the less aggressive forms of intervention might be too subtle to be noticed or effective. The least aggressive interventions, such as nonforceful touching, might simply be insufficiently forceful to get noticed by an antagonist engaged in a physical fight. However, if an intervening third party wants to influence an antagonist that has performed few or no violent behaviors these milder and almost symbolic interventions might suffice. Following this, we expect third parties to intervene more aggressively when the preceding level of aggression by the antagonist is high as a way to forcefully change the course of the conflict.

The second way the behavior of an antagonist might influence the level of aggression of a third-party intervention is when a third-party intervenes in a conflict to punish an antagonist for wrongdoing (Tedeschi & Felson, 1994). Here, the third party is not using aggression to influence the trajectory of the conflict, but rather to make things right. The antagonist has—from the perspective of the third party—overstepped some boundaries and must be punished for these transgressions. We expect that a high preceding level of aggression by the antagonist will engender more aggressive interventions by the third party, since it seems reasonable to assume that the larger the transgression, the harsher the punishment.

The third way the behavior of an antagonist might influence the aggression of a third-party intervention is through emotional contagion. Emotional contagion is “the tendency to automatically mimic and synchronize expressions, vocalizations, postures, and movements with those of another person’s and, consequently, to converge emotionally” (Hatfield et al., 1993, p. 96). Emotions thus rub off on individuals that are interacting with each other (Collins, 2014). A recent metareview has argued that emotional contagion is a central factor influencing when third parties intervene in interpersonal conflicts (Fischer et al., 2011). No research has, to our knowledge, looked into how emotional contagion might influence the aggressiveness of an intervention. However, following the definition of emotional contagion it seems plausible that if aggression is contagious, then more aggressive expressions of emotion by an antagonist will engender more aggression by the intervening third parties. Following this third path of influence we thus again expect that third parties will intervene more aggressively when the preceding level of aggression by the antagonists is high.

All three paths through which antagonist behavior might influence the level of aggression of third-party interventions thus predict a positive correlation between the two: a higher level of aggression by the antagonists will engender more aggressive intervention behaviors by the third parties. This pattern is supported by an analysis of third-party aggression on the situational level by Parks et al. (2013). They argue that more aggressive and dangerous situations increase the likelihood that third parties will intervene aggressively (Parks et al., 2013). This study, however, does not take the development of the situation into account, but rather measures the level of aggression as a situational characteristic.

To sum up, in order to investigate the nature of third-party aggression in interpersonal conflicts, the two-part research question of this article is as follows: *Do third parties change the level of aggression of their interventions throughout a conflict?* And if so, *are these changes in level of aggression of third-party interventions shaped by the aggressiveness of the targeted antagonist?*

## Data and Methods

### Collecting the Video Footage

The analysis is based on CCTV footage of interpersonal conflicts from Amsterdam. The authors were granted access to CCTV files by the Dutch ministry of justice and the footage was collected in collaboration with the Amsterdam Police Department and the Municipality of Amsterdam. The conflicts were recorded by camera operators watching the live-streaming footage 24 hours a day, 7 days a week. The data collection began in April 2017 and ended in August the same year. The CCTV cameras are located throughout the city of Amsterdam on streets and squares that the Mayor of Amsterdam’s office has identified as hot spots of crime and disorder.

As part of their usual working practice, the operators record any kind of violent conflict. In addition to their usual practices, we instructed the operators to record nonviolent conflicts. We instructed the operators to keep an eye out for behavioral indicators such as people having heated arguments, pushing and/or pulling each other, taking of their shirts or jumpers, and restless groups of people. We furthermore instructed the operators to collect as much footage as possible of the involved parties before and after the conflicts.

### Definition of a Third Party

In this study, we do not conceptualize being a third party as a situationally fixed role, but rather as a type of behavior. For a behavior to qualify as an intervention behavior in the analysis it must be performed by individual A (third party) toward individual B (Antagonist 1) who is engaged in a conflict with individual C (Antagonist 2). This classification is made irrespective of whether individual A has previously been directly involved in the conflict as an antagonist, or not.

Since intervention is a type of behavior and not a situational role, the same individual can initially intervene as a third party and later become an antagonist, or vice versa. Previous studies exemplify that the ascribed roles in conflict situations are dynamic and oftentimes change throughout the situation. Luckenbill (1977) has argued that categories such as victim and perpetrator are “heuristic labels” that might change throughout the conflict. Similarly, Felson et al. note that “in about half the cases where third parties are active (48 percent), third parties were originally one of the main antagonists and either the victim or offender interceded” (Felson et al., 1984, p. 457). Based on these insights, we find it preferable to classify each behavior according to what role it has in the situation, rather than classifying each individual.

### Selection Criteria

In this article, we investigate the consistency in aggression of third-party interventions. To do this, we record all intervention behaviors across the videos where the preceding behavior by the same individual is also an intervention behavior. In other words, the units of analysis are all interventions that are not an individual’s first intervention in the conflict. The exclusion of the first intervention was necessary because we need at least two behaviors per individual to investigate the consistency of their behavior. This implies that individuals who only made a single intervention were excluded.

In total, we collected CCTV footage depicting 165 conflict situations. We audited each recording for its utility for the study. Only files that conform to the following criteria are part of the final sample:

1. An interpersonal conflict is visible in the recorded footage
2. The quality of the video (resolution, brightness, and frames per second) is sufficiently high to allow the coding
3. There are no or only negligible breaks in the recording
4. There is at least one third party performing two consecutive intervention behaviors in the conflict

Out of the original sample of 165 situations, 25 of the videos did not depict a conflict, 36 of the videos were too low resolution to be coded, and 72 of the videos had substantial parts of the conflict missing (the categories are not mutually exclusive). Another 16 of the remaining 62 situations only had third parties who intervened only once or not at all, resulting in a final sample of 46 situations. 28 of the intervention behaviors in the material were directed toward more than one antagonist at the same time. These interventions were excluded from the material.

**Figure 1.**
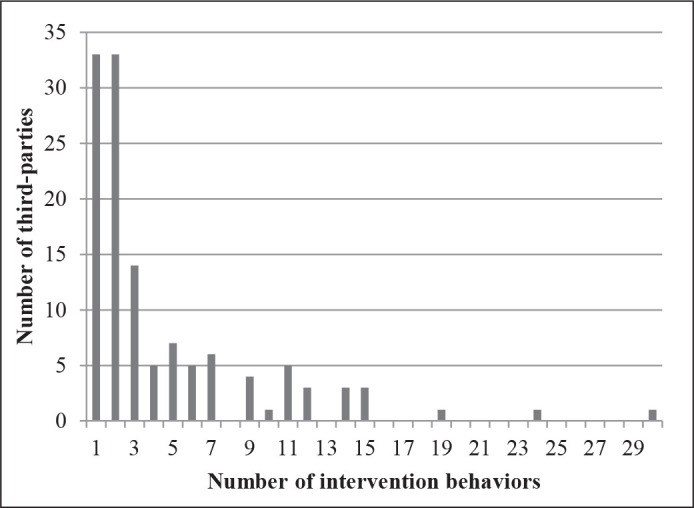
Number of third-parties by number of intervention behaviors they perform (*N* = 125).

The final sample comprises 46 situations containing 125 third parties performing 565 intervention behaviors where their immediately preceding behavior was another intervention behavior. Figure 1 shows the distribution of interventions per third party. The majority of observed individuals perform either 1 or 2 intervention behaviors^1^ and the number of individuals decreases as the number of intervention behaviors increases. The highest number of interventions by the same individual is a staggering 30.

### Coding the Video Footage

The CCTV clips were coded using BORIS (Behavioral Observation Research Interactive Software) (Friard & Gamba, 2016). This program allows users to simultaneously watch the CCTV footage and code the observed behaviors. The program adds a timestamp for each code corresponding to the time the behavior occurs in the observed footage, which allows us to keep the chronology of the observed behaviors. We code the actor and a target of each behavior and whether the behavior is an intervention or a conflict behavior.

The variables of this study are based on a coding scheme (Appendix 1) detailing definitions of the coded conflict behaviors. The coding scheme was developed by watching a subsample of the collected footage numerous times and after reviewing other coding schemes used to analyze antagonist and third-party behaviors (Liebst et al., 2018; Lindegaard et al., 2017; Philpot, 2017).

### Measurement

The dependent variable of the study measures the level of aggression of each intervention behavior in the conflict situations (where the preceding behavior by the same individual is also an intervention behavior). To code the level of aggression of these interventions, we use the scale of aggression developed by Parks et al. (2013). While this scale originally has eight levels (0-7), we reduced the number of levels to three. We did this to reduce the complexity of the measure and because the videos do not contain sound which makes some of the levels obsolete. Table 1 summarizes the three levels of aggression used in this study, their corresponding levels in the original eight-level scale by Parks et al. (2013), and the corresponding behaviors from the coding scheme. The three-level scale used for this study spans from *low aggression* (soft and nonaggressive intervention behaviors), over *medium aggression* (aggressive but nonviolent behaviors), to *high aggression* (violent behaviors).

**Table 1.** Scale of Levels of Aggression and the Corresponding Behaviors.

Level of Aggression	Corresponding Levels on Parks et al. (2013) Scale	Behaviors
Low	0, 1	Nonforceful touching and calm hand gestures
Medium	2, 3, 4, 5, 6	Holding back, blocking, hauling a person off, push, aggressive gesturing, and invading space
High	7	Kicking, hitting, striking with an object, throwing or aggressive pulling, and wrestling or grappling

The first independent variable measures the level of aggression of the intervention behavior that precedes the dependent variable. This variable thus measures the level of aggression by the third party before the intervention recorded by the dependent variable and thereby allows us to investigate whether the intervention has changed in aggression or remained the same. To code this variable, we used the same aggression scale as used for the dependent variable. The second independent variable of the study measures the cumulative number of violent behaviors (the high level on the aggression scale presented in Table 1) by the antagonist at the time of intervention.

### Control Variables: Social Relationship and Gender

We also code two control variables based on the video footage: the gender of the third party and social relationships between the antagonists and the third parties. We include the two control variables because previous studies find they influence the likelihood that a third party will intervene aggressively (Parks et al., 2013; Phillips & Cooney, 2005; Tedeschi & Felson, 1994). While these factors are not of primary interest to the research questions of this article, we include them in the analysis to avoid bias by omitting relevant variables.

We code the gender of the individuals observed in the footage based on their clothes, facial features, hair, and body type. We infer the social relationships of the involved parties of the conflict based on the observed tie signals among the actors in the footage. The visual apparency of social relationships has been described by Goffman (1971) and Hall (1966) who argue that the physical proximity between individuals in public spaces correlates with the social proximity of the individuals. This has later been corroborated in empirical studies observing pedestrian behavior (Ge et al., 2012; McPhail & Wohlstein, 1982; Solera et al., 2013) and in conflict situations (Liebst et al., 2018).

Since most of the videos include footage of the antagonists arriving at and leaving the conflict, we use this information as a cue of a social relationship when it was available. If two individuals arrive at or leave the scene in proximity to each other, this is taken as an indicator that they have a social relationship. Furthermore, we also draw on other social signifiers such as groups wearing matching clothes or uniforms, standing close to one another, being engaged in casual conversation, holding hands, or similar signs when we assess the social relationships. In this study, we do not discriminate between different kinds of social relationships and all relationships are assumed to be symmetrical, so that if person A has a social relationship to person B, person B also has a social relationship to person A.

### Assessment of Reliability

The CCTV footage was encoded by the P.E. of this article. In order to estimate the reliability of the encoding of the videos a trained graduate student independently coded approximately 20% of the material. Any disagreements between the coders were resolved prior to the analysis. We calculated Cohen’s Kappa (κ) to estimate the extent of agreement in the double coded situations. In order to make the codes comparable, each individual in each situation was given a unique identifier to allow both coders to identify the same individual in the videos. Agreement was defined as both coders identifying the same type of behavior performed by the same actor toward the same target within a one-second window. Following the literature on interrater reliability we calculate the agreement for the measures as they are used in the analysis (Krippendorff, 2004). All three levels of aggression have an interrater reliability that falls within the “moderate” or “substantial” agreement (*κ*_low aggression_ = 0.539, *κ*_medium aggression_ = 0.618, and *κ*_high aggression_ = 0.671) and the interrater reliability scores for gender and social relations are almost perfect (*κ*_gender_ = 1 and *κ*_social relation_ = 0.89) (Landis & Koch, 1977).

### Estimation Methods

In the analysis we use a hierarchical, multinomial logistic regression to estimate the model. The strength of the multinomial model is that it allows us to estimate a logistic regression with a dependent variable that has three outcomes rather than the usual two. This is necessary in this study because the dependent variable measures the three levels of aggression of the third-party interventions. We use a multilevel model in order to take into account that we have multiple observations for some of the third parties. In order to take into account that the observations are nested in situations (because some conflict situations involve multiple intervening third parties), we estimate the model with cluster corrected standard errors. We run the model in STATA 14 using the GSEM package.

## Results

### Descriptive Statistics

Table 2 shows the descriptive statistics for all the variables of the analysis. The dependent variable measures the level of aggression of intervention behaviors where the immediately preceding behavior by the same individual was also an intervention behavior. This variable is an ordinal variable with three outcomes. The table shows that 25% of the intervention behaviors are on the lowest level of aggression, 71% are on the medium level of aggression, and just 4% are on the highest level of aggression.

The first explanatory variable (Q1) measures the level of aggression of the intervention behaviors that precedes the dependent variable. As shown in Table 2, 24% of the preceding intervention behaviors are on the low level of aggression, 73% are on the medium level of aggression, and only 4% are on the highest level of aggression.

**Table 2.** Descriptive Statistics of Intervention Behaviors (*N* = 565).

	Variable	Mean	Std. Dev.	Min	Max
Dependent variable	Aggression level of intervention behavior:				
	• Low	0.25	0.44	0	1
	• Medium	0.71	0.46	0	1
	• High	0.04	0.19	0	1
Hypothesized explanatory variables	(Q1) Aggression level of the preceding intervention behavior:				
	• Low	0.24	0.42	0	1
	• Medium	0.73	0.45	0	1
	• High	0.04	0.19	0	1
	(Q2) No. of aggressive behaviors by antagonist	1.70	2.36	0	12
Control variables	Social relationship	0.49	0.50	0	1
	Female third-party	0.19	0.40	0	1

The second hypothesized explanatory variable (Q2) measures the number of violent behaviors performed by the antagonist before being targeted with the intervention behavior. The highest number of violent behaviors performed by an antagonist in the empirical material is 12 behaviors. The lowest is none. On average, the targeted antagonists have performed 1.7 violent behaviors prior to being targeted with the intervention behavior.

The last two variables in Table 2 are the control variables. The first control variable is the social relationship between the third party and the antagonist targeted with the intervention behavior (the dependent variable). This variable shows that 49% of the third parties have a social relation to the person they target. The second control variable is a dummy designating the gender of the third party performing the intervention. Table 2 shows that the majority of interventions (81%) in the material are performed by men and only one in five (19%) are performed by women.

Table 3 shows the transitions in aggression of the coded intervention behaviors. In this table, the rows denote the intervention behaviors immediately preceding the intervention behaviors in the columns, and the columns denote the intervention behaviors that follow those in the rows. The columns and rows are thus the dependent variable and the first independent variable (Q1) presented in Table 2, respectively.

**Table 3.** Transition Matrix of Intervention Behaviors (First Behavior) and the Subsequent Behavior by the Same Individual (Second Behavior) (*N* = 565).

Level of aggression of preceding intervention behavior (first behavior)	Level of Aggression of Intervention Behavior With a Preceding Intervention Behavior (Second Behavior)			
	Low	Medium	High	Total
Low	64	68	1	133
Medium	77	321	13	411
High	3	10	8	21
Total	144	399	22	565

The transitions to the same level of aggression are found on the diagonal of Table 3. The table thus shows that approximately 70% of the coded intervention behaviors (393 observations) are preceded by an intervention behavior on the same level of aggression. Two consecutive interventions are thus typically on the same level of aggression.

Among the remaining approximately 30% of the transitions in the empirical material there are only 4 observed cases of intervention behaviors that are followed by an intervention behavior two levels of aggression above or below the first behavior. The remaining 29.5% of intervention behaviors are followed by a behavior that is either one level above or below the level of aggression of the preceding intervention. Table 3 thus shows that while most intervention behaviors are followed by equally aggressive behaviors, more than a quarter are not, and these shifts indicate a change in intervention behavior. This is similar to what have been observed in previous studies (Levine et al., 2011; Liebst et al., 2019a).

### Adaptive Intervention Behavior

The aim of the analysis is to investigate whether the behavior of the antagonists influences the level of aggression of the intervention behaviors of third parties when the preceding behavior by the third party is taken into account. We investigate this using a multilevel, multinomial logistic regression with cluster corrected standard errors. We use a multilevel model to account for how some third parties perform multiple interventions and cluster corrected standard errors to account for how some conflicts involve multiple third parties.

Table 4 shows the results from the analysis. The table is divided in two overall sections; Section (1) and (2). The first section shows the logistic estimation of the likelihood that the intervention behavior will be on the low level of aggression and the second section shows the likelihood that an intervention behavior will be on the high level of aggression. The reference category, and thus the level of comparison, is the medium level of aggression.

The first section of Table 4 shows the results for the estimation of the likelihood that an intervention behavior is on the low level of aggression. The first explanatory variable is the number of violent behaviors performed by the targeted antagonist prior to the intervention. The estimated odds ratio is 0.79 and is statistically significant (*p* = .001). This means that an increase in the number of violent behaviors by the targeted antagonist engenders a reduced likelihood that the intervention behavior will be on the low level of aggression. Each additional violent behavior by the targeted antagonist thus reduces the odds of a low-aggression intervention by a factor .79. This means that three violent behaviors by the antagonist reduce the odds of a low-aggression intervention to half ((.793)^3^ = .50) and 12 violent behaviors (which is the highest observed number in the videos) is equal to an odds ratio of 0.06 ((.793)^12^ = 0.06).

The second variable in the table is the binary variable measuring whether the preceding intervention behavior was on the low level of aggression. This independent variable is a statistically significant predictor (*p* < .005) and has a medium effect size with an odds ratio of 2.4 (Sullivan & Feinn, 2012). This shows that when the preceding behavior is on the low level of aggression the odds is 2.4 times larger than the subsequent behavior will be on the same level of aggression. The variable measuring if the previous behavior was on the high level of aggression is not statistically significant. There is thus not a statistically significant difference between the odds that an intervention behavior on the low level of aggression was preceded by a behavior on the high level compared to the reference category (the medium level of aggression). None of the control variables are statistically significant in the estimation of the likelihood that the following behavior is on the low level of aggression.

**Table 4.** The Results of the (Hierarchical) Multinomial Logistic Regression (*N* = 565).

Outcome on Dependent Variable	Variable	*B*	Robust *SE*	*Z*	*p* Value	*OR*
(1) Low aggression	No. of aggressive behaviors by antagonist	–0.232	0.073	–3.190	.001	0.793
	Preceding intervention behavior: low aggression	0.855	0.304	2.820	.005	2.351
	Preceding intervention behavior: medium aggression (reference)	0				1
	Preceding intervention behavior: high aggression	0.643	0.811	0.790	.428	1.903
	Social relationship	–0.113	0.255	–0.440	.659	0.894
	Female	–0.496	0.392	–1.270	.206	0.609
(2) High aggression	No. of aggressive behaviors by antagonist	0.360	0.091	3.940	<.001	1.434
	Preceding intervention behavior: low aggression	–1.139	1.066	–1.070	.286	0.320
	Preceding intervention behavior: medium aggression (reference)	0				1
	Preceding intervention behavior: high aggression	2.025	0.700	2.890	.004	7.573
	Social relationship	–1.971	0.848	–2.330	.020	0.139
	Female	–0.099	0.674	–0.150	.883	0.905

The second section of Table 4 shows the model for estimating the likelihood that an intervention behavior is on the high level of aggression. The first variable in this section is the number of violent behaviors performed by the targeted antagonist prior to the intervention. This variable is statistically significant (*p* < .001). The odds that an intervention behavior is on the high level of aggression is thus 1.26 higher when the targeted antagonist has performed one violent behavior (this variable is a count variable and when the targeted antagonist has performed more aggressive behaviors this factor will be more influential).

The second variable indicates when the preceding intervention behavior is on the low level of aggression. This variable is not statistically significant. There is thus not a statistically significant difference in the odds that an intervention behavior on the high level of aggression was preceded by a behavior on the low level compared to the reference category (the medium level of aggression).

The third variable in the second section of Table 4 is a binary variable measuring if the previous intervention behavior by the same third party was on the high level of aggression. This variable is statistically significant (*p* = .004) and has a very large effect size with an odds ratio of 7.5 (Sullivan & Feinn, 2012). It is thus apparent that when the preceding intervention is on the high level of aggression the odds that the subsequent behavior will be on the same level is more than seven times larger.

The control variable measuring if the third party and the antagonist targeted with the intervention behavior are from the same social group is also statistically significant (*p* = .019) and has a very large effect size (0.13). It is thus much less likely, everything else being equal, that an intervention behavior will be on the high level of aggression if the third party has a social relation to the target of the intervention. The gender of the third party is not statistically significant. We thus do not find a difference between men and women in the likelihood that the intervention behavior will be one the high level of aggression.

## Discussion

This study investigated the changes in aggression of third parties intervening into interpersonal conflicts. This study contributes to our understanding of interpersonal violence, since previous research has shown that the aggression of third-party intervention determines how the intervention influences the development of the conflict. Understanding how situational factors influence the aggression of intervention is thus a key aspect of understanding when interpersonal conflicts escalate. This study shows for the first time how the aggression of intervention is not always fixed, but rather something that can change throughout the situation and is influenced by the behavior of the antagonists.

Based on the previous research we formulated a two-stage research question: First, we asked if third parties intervene in a consistent manner throughout a conflict situation. We investigated the consistency of the intervention of third parties in two different ways. First, we constructed a transition matrix of the actual transitions between the different levels of aggression in two consecutive intervention behaviors performed by the same third party. Here, the overall pattern was that an intervention behavior typically is followed by another behavior on the same level of aggression. However, while this was the overall trend, this analysis also showed that 30% of the observed intervention behaviors are preceded by an intervention on a different level of aggression.

Second, and to further qualify this initial finding; we estimated a multivariate model to see if the preceding behavior was an influential predictor when other aspects of the situation were taken into account. The multivariate statistical model corroborated the findings from the transition matrix and showed that the consistency assumption of the scientific literature has some warrant. The analysis thus shows that third parties, everything else being equal, are more likely to intervene at consistent levels of aggression. The consistency in the level of aggressive behavior shows that the mutually exclusive categorizations of third parties typically used in the empirical literature – such as *aggressive* vs. *nonaggressive* (Wells & Graham, 1999), *mediator* vs. *partisan* (Cooney, 1998), and *mediate* vs. *engage* (Felson et al., 1984)—appear to fit the majority of behavioral transitions in the empirical material.

Furthermore, the transitional matrix showed that there are few observed radical changes in aggression. While 3 out of 10 intervention behaviors follow an intervention behavior on a different level of aggression, these changes are typically only slightly more or less aggressive than the preceding behavior. This pattern was, however, not corroborated in the multivariate model. In the statistical model, we found that only preceding behaviors on the same level influenced the subsequent behavior. The model, thus, did not find a significant difference between the likelihood that intervention behaviors on the low and medium level of aggression are followed by an intervention on the high level of aggression.^2^

While the majority of the intervention behaviors conform to the expectation of consistency, we also found that the third parties mirror the aggressiveness of the antagonists. The multivariate model shows that an intervention toward an antagonist who has been very aggressive prior to the intervention is more likely to be more aggressive as well, even when the preceding behavior of the third party is accounted for. Inversely, an increase in the number of violent behaviors by the targeted antagonist reduces the likelihood that the intervention behavior will be on the low level of aggression.

Just as previous research has found that the dangerousness of a situation influences how likely it is that a third party will intervene (Fischer & Greitemeyer, 2013), this study finds that the dangerousness also influences *how* a third party intervenes. An antagonist who has performed more violent behaviors will be targeted with more aggressive interventions—third parties fight fire with fire. This influence of the behavior of the antagonist shows how there is a bidirectional influence between the aggressiveness of the intervening third parties and the antagonists of the conflict. Previous research has shown that the level of aggression of intervention behaviors impacts the aggressiveness of the antagonists (Levine et al., 2011; Parks et al., 2013; Phillips & Cooney, 2005). This article shows that the opposite is true as well: The aggressiveness of the antagonist influences the level of aggressiveness of the intervention behaviors.

This interconnectedness corroborates the importance of drawing on an interactionist framework in the analysis of third-party interventions. While this perspective previously has been used to study the antagonists of interpersonal conflicts in the scientific literature, this study shows how a similar framework is beneficial in the study of third-party behaviors. The interactionist conception of third-party behavior allows us to see that these behaviors are not only predetermined qua individual background of the third parties, but also adapted to the behavior of the other people in the situation the third parties are responding to the development of conflict situations.

The bidirectional influence between the aggressiveness of antagonists and third-party interventions implies that the third parties have a polarizing effect: In conflicts where the antagonists are not very aggressive, a third party will be more likely to intervene on a lower level of aggression. This intervention will—according to previous research (Levine et al., 2011; Parks et al., 2013; Phillips & Cooney, 2005)—be more likely to de-escalate the conflict and placate the antagonists further. Conflicts with very aggressive antagonists will inversely increase the likelihood that third parties will intervene more aggressively and in turn increase the risk that the conflict will escalate even further.

This polarizing effect has implications for both real-life conflict prevention and scientific research. While third parties are a potential source of violence prevention (Levine et al., 2011; Liebst et al., 2019b), this study shows that third parties might be best at deescalating the less aggressive conflicts where they are the least likely to act aggressively themselves. This could imply that the severe conflicts are better left with the professional interveners—such as policemen or security personnel—that have the training and experience to handle these stressful and dangerous situations.

Bystander intervention programs can overcome this polarizing effect in two ways. The first way is to implement an upper limit of severity after which lay-persons are recommended to search for a professional rather than take action themselves. The other option is to inform third parties about the danger of mirroring the aggression of the antagonists and the necessity of remaining calm in heated conflict situations, even though their first impulse might be otherwise. Both of these options have their shortcomings. The first, because formal guardians are rarely readily available in the conflict situations and the second because this kind of self-control probably requires training and experience that is beyond most lay-persons.

The analysis shows that a social relationship between the intervening third party and the antagonist decreases the likelihood of the third party becoming very aggressive. Previous research argues that the degree of intimacy might inhibit the use of violence out of fear for the consequences this might have on the relationship (Tedeschi & Felson, 1994). This finding indicates that this group might be worth targeting specifically in violence prevention programs. The previous research has found that third parties take responsibility for the actions of their peers and that people with social relationships have “handles” on antagonists that allow them to more effectively influence their behavior (Ejbye-Ernst et al., 2020; Felson, 1995). The current research adds to this, by showing that this group also has a lower risk of becoming violent themselves and thus potentially escalating the conflict further.

The findings of this study also have implications for the study of interpersonal conflicts in general. The interconnectedness between the behaviors of the antagonists and third parties of the conflicts shows the necessity of looking at the entire social context when studying interpersonal conflict or violence. The behavior of each of the individuals in the conflict depends on the preceding behavior by everyone in the situation. This means that we cannot understand the violence of an antagonist without looking at the preceding behavior of third parties, but also, that we cannot understand the behavior of the third parties without taking the behavior of the preceding antagonist into account. Isolating one part of this system means only getting half the story.

The bidirectional relationship between the aggressiveness of the antagonists and the intervention behaviors of third parties also has implications for future research. This is especially pertinent for studies investigating the effect of third-party interventions. These studies have typically assumed that third-party behavior is constant (Parks et al., 2013; Phillips & Cooney, 2005) and thus overlook that the aggressiveness of third-party intervention is shaped by the aggressiveness of the antagonists. Future inquiries investigating the effect of third-party interventions must account for this feedback effect. Any inquiry that does not account for the bidirectionality in some way will be left guessing whether a correlation between intervention behavior and conflict development is due to the intervention behaviors influencing the conflict or the third party adjusting their behavior to the conflict. This means that using situationally fixed roles, such as aggressive and nonaggressive third parties, do not allow researchers to see the complex interactions and developments that arise throughout the conflict situations. Based on the findings of this study, future research on interpersonal conflicts should therefore allow for third parties to change between different roles as they react to the conflict development.

The study faces three limitations. First, the lack of sound on the videos might have impacted the categorization of the behaviors according to aggression. The scale used to categorize the aggressiveness of the behaviors in the videos was reduced in complexity in this study from the original scale developed by Parks et al. (2013). This reduction in complexity was necessary because the videos lack audio. This means that we can only observe the behaviors of the involved parties, but are left at a loss when it comes to the content of the conflicts and the verbal acts that might take part during the conflict.

Second, the sole reliance on CCTV footage limits the investigation to behavioral aspects of the conflicts. The CCTV footage allow us to view the behavior of the conflicts in very fine detail, but it leaves us empty handed when it comes to the feelings, thoughts, and motivations of the involved parties. This is a limitation for this study since the motivation might be a central factor in whether third parties are influenced by situational changes or not.

Third, while the use of CCTV footage offers insights into the development of interpersonal conflicts that are difficult to reach through conventional methods, the recording of conflicts through CCTV cameras might be limited or biased in certain ways. The conflicts under scrutiny in this study all happen in public spaces. This means that conflicts that are confined to private spaces are outside the scope of this study. Furthermore, the data might be influenced by a latent bias in what constitutes a potential conflict situation. Latent ethnic and racial biases among police officers has received much attention (Antonopoulos, 2003; Engel et al., 2002), and we cannot rule out that similar biases might influence the gaze of the operators recording the conflict situations for this study. We tried to counteract this potential bias by supplying the operators with a list of behavioral indicators that a conflict was emerging (as described in the methods section).

## Appendix

**Appendix 1:** Behavioral Coding Scheme

Behavior	Brief Definition	Level of Aggression
Calming hand gestures	Slow, calming gestures performed with open hands usually with the palm of the hand facing the ground or directed toward the receiver. Actors gesticulating with their hands while talking should only be coded if the gestures in themselves seem to be calming. Not all slow gestures are thus calming hand gestures.	Low
Aggressive gestures	Fast, angry, and expressive gestures. Aggressive gestures typically involve pointing at someone in a forceful manner, palms turned upwards, simulating hitting or slapping, movements that incite the other party to attack (e.g., waving them closer). Aggressive gestures also include hitting objects.	Medium
Invading space	The actor moves his face very close to the face of the receiver without touching him/her. This usually involves just a few centimeters of distance between the actor and receiver, but could be slightly more.	Medium
Nonforceful touching	Stroking or gently touching the receiver without physically holding him/her back or trying to move him/her in a particular direction.	Low
Blocking or holding a person back	Either blocking an antagonist from crossing a specific point or holding on to an antagonist trying to fixate them at a specific point.	Medium
Hauling a person off	The actor is actively trying to change the course, position, path, or direction of the receiver by holding on to the receiver and (attempt to) lead, pull or carry that individual in some direction.	Medium
Throwing or aggressively pulling a person	A forceful and fast paced pull where the actor grips the receiver and throws or aggressively pulls them. The actor will typically try to forcefully move the receiver of the act while the actor remains more or less in the same spot.	High
Push	The actor uses his or her arms, chest or shoulder to increase the distance between the actor and the receiver or push the receiver sideways.	Medium
Hitting	The actor hits the receiver with a clenched or open hand. A hit is when the actor uses his/her hand to strike someone else with relative high velocity.	High
Striking with object	The actor uses an object to strike the receiver either by hitting them or throwing the object at them.	High
Kicking	Kicking the receiver with foot or knee. The actor uses his/her foot or leg to strike the receiver.	High
Wrestling/grappling	Grappling/wrestling is a behavior seen when the actor and receiver are in close combat. Grappling/wrestling is characterized by the actor holding onto, shaking, moving, or struggling with a receiver often in a chaotic and messy fashion.	High

## Notes

1. As explained, this is excluding each individual’s first intervention in the conflict, because the first intervention cannot be compared with a previous intervention.

2. Note, however, that the data poses a structural constraint on big changes since the high aggression levels are very rare. This means that the statistical test of the radical transitions might be a product on too few observations of these transitions. This result should thus be interpreted with caution.

## Author Biographies

**Peter Ejbye-Ernst** is a PhD and MSc candidate at the Netherlands Institute for the Study of Crime and Law Enforcement (NSCR) and the Department of Sociology at the University of Amsterdam. His interests include interpersonal conflicts, microsociology, social control, and systematic behavioral analysis.

**Marie Rosenkrantz Lindegaard** is a PhD professor of sociology at the University of Amsterdam and a senior researcher at the Netherlands Institute for the Study of Crime and Law Enforcement (NSCR). Her research interests include interactional aspects of offending, victimization, guardianship and law enforcement, street culture, observational methods, and urban ethnography in South Africa.

**Wim Bernasco** is a PhD senior researcher at the Netherlands Institute for the Study of Crime and Law Enforcement (NSCR) and a professor in the Department of Spatial Economics at the Vrije Universiteit Amsterdam. His interests include offender travel behavior, target selection, situational causes of offending and victimization, and research methodology.
